# Application of a novel 4.5F electrohydraulic lithotripsy probe for complex biliary stone fragmentation: a case series

**DOI:** 10.1016/j.igie.2025.12.009

**Published:** 2025-12-29

**Authors:** Kanika Garg, Thomas J. Wang, Agnieszka Maniak, Christopher G. Chapman, Irving Waxman, Ajaypal Singh, Neal A. Mehta

**Affiliations:** Division of Digestive Diseases and Nutrition, Center for Interventional and Therapeutic Endoscopy, Rush University Medical Center, Chicago, Illinois, USA

## Abstract

**Background and Aims:**

Large biliary stones (>25 mm) present significant therapeutic challenges, with standard endoscopic techniques frequently failing to achieve complete ductal clearance. Conventional 3F electrohydraulic lithotripsy (EHL) probes often require multiple sessions or result in incomplete fragmentation. We present a novel approach using a 4.5F EHL probe (Walz Elektronik GmbH, Rohrdorf, Germany) for successful management of large refractory biliary stones.

**Methods:**

Three female patients (aged 57, 59, and 67 years) with large biliary stones (35-45 mm) previously refractory to standard techniques underwent direct cholangioscopy with a 4.5F EHL probe fragmentation using an 11F cholangioscope (eyeMAX; Micro-Tech, Ann Arbor, Mich, USA). The 4.5F probe delivers energy up to 950 mJ with greater surface area contact than standard 3F probes.

**Results:**

Complete ductal clearance was achieved in all 3 cases within a single session, with successful fragmentation where conventional techniques had failed. Procedure durations ranged from 49 to 94 minutes. No adverse events occurred in any case.

**Conclusions:**

The 4.5F EHL probe represents a significant advancement for managing large refractory biliary stones, potentially reducing repeat procedures and obviating surgical intervention.

## Introduction

Large biliary stones exceeding 25 mm in diameter represent one of the most challenging scenarios in therapeutic endoscopy. Standard endoscopic approaches, including extraction balloons, retrieval baskets, and mechanical lithotripsy, frequently fail to achieve complete ductal clearance in these cases.^1^^,^^2^ Conventional electrohydraulic lithotripsy (EHL) using 3F probes through digital single-operator cholangioscopy has become the standard approach for fragmentation of large stones. However, the limited surface area and energy distribution of 3F probes frequently necessitate multiple sessions or result in incomplete fragmentation, particularly for stones exceeding 30 mm.^3^^,^^4^

We present 3 cases demonstrating the successful application of a novel 4.5F EHL probe (Walz Elektronik GmbH, Rohrdorf, Germany) through an 11F cholangioscope (eyeMAX; Micro-Tech, Ann Arbor Mich, USA) for complete fragmentation of large treatment-refractory biliary stones. Compared with standard probes, the 4.5F device provides greater surface area contact, delivers energy up to 950 mJ, and enables direct stone contact without a saline cushion requirement.

## Methods

This study was approved by the Institutional Review Board (#22110804). Three patients with large biliary stones refractory to conventional endoscopic techniques underwent cholangioscopy-guided EHL using the 4.5F probe. All procedures were performed with the patient under general anesthesia with direct cholangioscopic visualization using the 11F cholangioscope. All procedures used a single 4.5F EHL probe at maximum energy setting (intensity C, 950 mJ) for optimal stone fragmentation. Complete procedural details, including energy settings, duration, and outcomes, were systematically recorded. Procedure duration was defined as the time from scope insertion to scope withdrawal (“scope-in to scope-out” time).

## Case presentations

### Case 1

A 59-year-old female patient with a medical history of diabetes, as well as a history of complicated open cholecystectomy and postoperative external biliary drain placement initially, presented to an outside hospital with persistent right-sided abdominal pain with associated nausea and vomiting. Magnetic resonance cholangiopancreatography revealed a 35-mm obstructing stone in the lower third of the common bile duct (CBD). An internal–external biliary catheter was placed at the initial facility. She was then transferred to a second outside hospital where 2 endoscopic retrograde cholangiopancreatography (ERCP) attempts with mechanical lithotripsy were unsuccessful in achieving stone clearance, with the most recent attempt occurring approximately 1 week before presentation.

**Figure 1:**
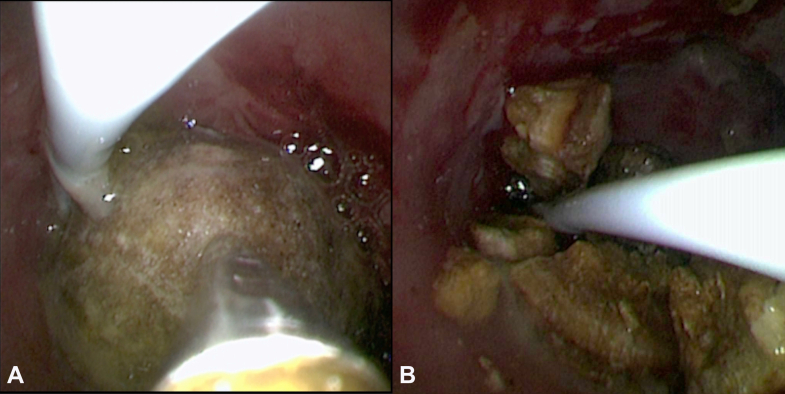
Case 1. **A,** Cholangioscopic view showing the 4.5F electrohydraulic lithotripsy probe positioned against a 35-mm stone before fragmentation. The white catheter represents an internal–external percutaneous biliary drain. **B,** Cholangioscopic view demonstrating successful stone fragmentation with multiple small fragments visible in the bile duct.

She then presented to our institution for further management. Laboratory values showed total bilirubin 0.5 mg/dL (normal, 0.2-1.3 mg/dL), alkaline phosphatase 230 U/L (normal, 30-125 U/L), aspartate aminotransferase (AST) 73 U/L (normal, 3-44 U/L), and alanine aminotransferase (ALT) 88 U/L (normal, 0-40 U/L). The patient underwent inpatient ERCP with cholangioscopy using the 11F cholangioscope. Previous sphincterotomy had been performed. Initial cholangiogram revealed severe biliary dilatation to 20 mm. The 11F cholangioscope provided excellent direct visualization of the stone's surface characteristics (Fig. 1A). Targeted fragmentation using the 4.5F probe at a 950-mJ setting systematically reduced the large stone into progressively smaller extractable fragments, with a procedure duration of 59 minutes (Fig. 1B). Balloon sweeping successfully removed all debris, with complete ductal clearance confirmed on final imaging. No adverse events occurred.

### Case 2

A 57-year-old female patient with no significant medical history initially presented the previous month with abdominal pain and jaundice caused by choledocholithiasis. Her first ERCP included sphincterotomy and stent placement, but multiple 20- to 30-mm distal CBD stones could not be removed. A subsequent ERCP revealed a 45-mm stone impacted near the cystic duct takeoff with upstream dilation. Balloon sphincteroplasty to 10 mm was performed, but mechanical lithotripsy failed. Cholangioscopy with conventional 3F EHL achieved only partial fragmentation. A plastic biliary stent was placed.

The patient presented approximately 7 weeks later for repeat outpatient ERCP with the 4.5F EHL probe to address the residual stone impacted near the cystic duct takeoff.

**Figure 2:**
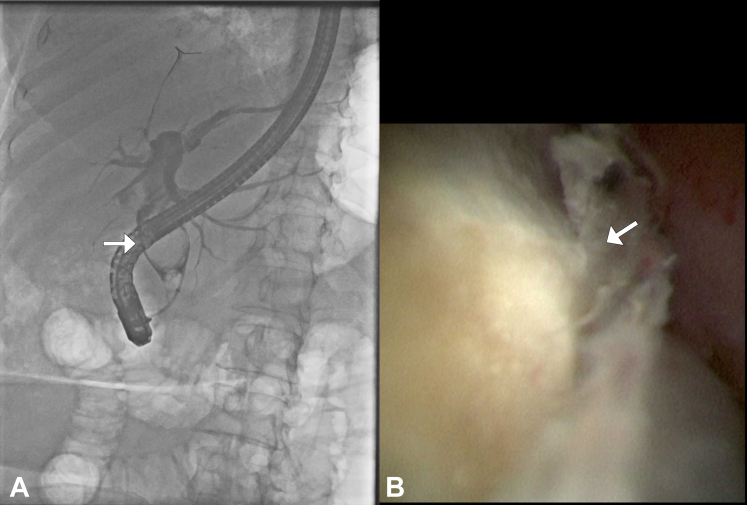
Case 2. **A,** Initial cholangiogram demonstrating a markedly dilated bile duct with a 45-mm stone impacted near the cystic duct takeoff (*arrow*). **B,** Cholangioscopic view of the obstructing stone (*arrow*) within the bile duct before fragmentation with the 4.5F electrohydraulic lithotripsy probe.

Laboratory values on the day of the 4.5F EHL procedure showed the following: total bilirubin, 0.5 mg/dL; alkaline phosphatase, 289 U/L; AST, 24 U/L; and ALT, 28 U/L. The patient underwent outpatient ERCP with cholangioscopy using the 11F cholangioscope. The markedly dilated bile duct measured 16 mm on cholangiogram (Fig. 2A). Under direct cholangioscopic visualization, the 4.5F EHL probe was positioned directly against the stone surface at a 950-mJ energy setting, permitting systematic fragmentation over a procedure duration of 49 minutes (Fig. 2B). Complete ductal clearance was achieved within a single session, with the final cholangiogram confirming the absence of residual filling defects. No adverse events occurred.

### Case 3

A 67-year-old female patient with a medical history of gastroesophageal reflux disease, obesity, arthritis, and prior cholecystectomy initially presented to an outside hospital with abdominal pain. Imaging revealed a 35-mm stone in the mid CBD with associated biliary ductal dilatation. She underwent ERCP at an outside hospital, where conventional 3F EHL achieved only incomplete stone removal.

**Figure 3:**
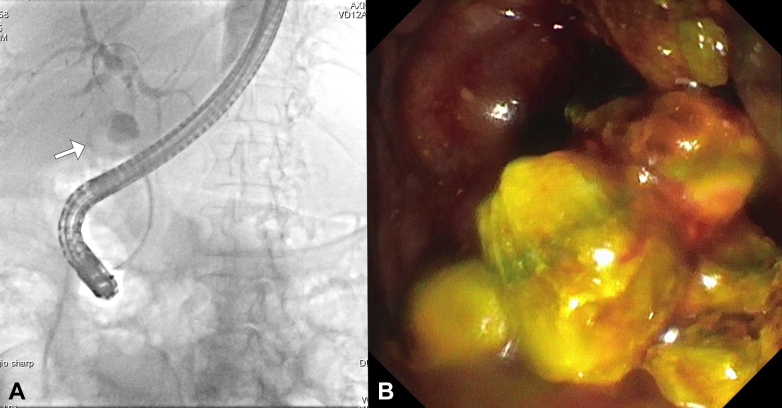
Case 3. **A**, Initial cholangiogram demonstrating a markedly dilated bile duct with a 35-mm stone in the midcommon bile duct (*arrow*). **B,** Endoscopic view of stone fragments being removed after successful fragmentation with 4.5F electrohydraulic lithotripsy probe.

She presented to our institution approximately 4 months later for repeat intervention to address the remaining stone. She was asymptomatic at that time. Laboratory values demonstrated as follows: total bilirubin, 0.3 mg/dL; alkaline phosphatase, 98 U/L; AST, 39 U/L; and ALT, 20 U/L. She underwent outpatient ERCP with cholangioscopy using the 11F cholangioscope. Previous sphincterotomy had been performed. Initial cholangiogram confirmed a markedly dilated bile duct to 20 mm with a large filling defect, consistent with a 35-mm stone (Fig. 3A). The 11F cholangioscope provided clear visualization and permitted precise targeting of the residual stone. The 4.5F probe delivered energy pulses directly to the stone surface at a 950-mJ setting, achieving complete fragmentation over a procedure duration of 94 minutes. Subsequent balloon extraction efficiently removed all stone debris from the biliary system (Fig. 3B). Final cholangiogram demonstrated complete ductal clearance with no residual filling defects. No adverse events occurred.

## Discussion

This case series demonstrates the clinical utility of the 4.5F EHL probe as a significant advancement in the management of large refractory biliary stones (Video 1, available online at www.igiejournal.org). In each case, previous attempts using standard endoscopic techniques, including mechanical lithotripsy and conventional 3F EHL, had failed to achieve complete ductal clearance. The enhanced capabilities of the 4.5F probe enabled successful single-session treatment where multiple previous interventions had been unsuccessful.

To our knowledge, this is among the first case series reporting use of a 4.5F EHL probe through an 11F digital single-operator cholangioscope for management of large biliary stones. Although conventional 3F EHL probes remain the standard approach, our experience suggests that large-diameter probes may offer advantages for particularly challenging stones that have failed standard techniques.

The superior fragmentation efficiency observed with the 4.5F probe likely results from several technical advantages over conventional 3F devices. The increased diameter provides greater surface area contact with target stones, enabling more efficient energy distribution. The device delivers the same adjustable energy levels up to 950 mJ (250-, 500-, or 950-mJ settings) but distributes this energy over a larger contact area without requiring a saline cushion, allowing direct stone contact for optimal fragmentation efficiency.^5^ Despite the larger diameter, the probe maintained excellent maneuverability through the 11F cholangioscope, permitting precise targeting in anatomically challenging locations such as the cystic duct takeoff region.

All procedures were performed under direct cholangioscopic visualization, which provided excellent stone targeting and enabled real-time assessment of fragmentation progress. The combination of enhanced visualization with the larger probe surface area contributed to the successful single-session outcomes in all cases. Notably, all procedures were completed without adverse events, demonstrating the safety profile of this approach.

Although no adverse events occurred in our series, the use of a larger-diameter probe theoretically carries potential risks that warrant consideration. The increased probe size could potentially cause greater mechanical trauma to the bile duct wall, higher risk of ductal perforation, or difficulty with maneuverability in smaller or more tortuous ducts. However, our experience suggests that when used with appropriate technique and direct visualization, these theoretical risks can be mitigated.

This enhanced performance may reduce the need for repeat procedures and decrease the likelihood of surgical intervention for complex biliary stone disease.^6^ The maintained maneuverability despite the larger probe diameter ensures that anatomically challenging locations remain accessible for treatment. The ability to achieve complete ductal clearance in a single session may improve patient outcomes while reducing health care costs associated with multiple procedures.

These technical innovations represent a valuable addition to the advanced endoscopist's therapeutic arsenal, particularly for managing complex biliary stone disease that has proven refractory to conventional approaches. The consistent success across cases with varying complexity and patient presentations suggests the broad applicability of this technique.

Several limitations of this case series should be acknowledged. The small sample size of 3 cases limits the generalizability of our findings, and larger studies will be needed to establish the broader efficacy and safety profile of the 4.5F EHL probe. In addition, this represents a single-center experience with a limited follow-up period, and long-term outcomes remain to be determined.

Our experience suggests the 4.5F EHL probe may be a useful option for managing large treatment-refractory biliary stones. We demonstrated superior fragmentation capability while maintaining excellent maneuverability, enabling successful single-session ductal clearance where conventional techniques have failed. This innovation may reduce repeat procedures, avoid surgical intervention, and provide enhanced treatment options for complex endoscopic stone management.

## Patient consent

The patients in this article have given written informed consent to publication of their case details.

## Disclosure

The following authors disclosed financial relationships: C. G. Chapman: Consultant for Boston Scientific, Olympus, Medtronic, Steris Endoscopy, AbbVie, and Phathom Pharmaceuticals. I. Waxman: Consultant for Boston Scientific, Cook Medical, and Medtronic. A. Singh: Consultant for Boston Scientific. N. A. Mehta: Consultant for Boston Scientific, Olympus, and Castle Biosciences. All other authors disclosed no financial relationships.

## Supplementary data

Video 1 Application of a novel 4.5-F electrohydraulic lithotripsy probe for fragmentation of large, treatment-refractory biliary stones through an 11-F cholangioscope in 3 patients.
